# Trans-oral Extra Tonsillar Approach of Styloidectomy for Treatment of Eagle’s Syndrome among Operated Cases of the Department of Otolaryngology-Head and Neck Surgery at a Tertiary Care Hospital: A Descriptive Cross-sectional Study

**DOI:** 10.31729/jnma.6756

**Published:** 2021-08-31

**Authors:** Deepak Regmi, Rachana Baidhya, Ashik Rajak, Sangita Shrestha, Meera Bista

**Affiliations:** 1 Department of Otorhinolaryngology-Head and Neck Surgery, Kathmandu Medical College Teaching Hospital, Kathmandu, Nepal

**Keywords:** *eagle's syndrome*, *stylohyoid ligament*, *styloidectomy*, *styloid process*

## Abstract

**Introduction:**

Eagle's syndrome is a poorly understood clinical entity that has variable presentations like recurrent throat pain or foreign body sensation, dysphagia, or facial pain. With a confirmed diagnosis, a surgical approach is considered appropriate for its treatment. This study aims to find out the prevalence of trans-oral extra tonsillar approach of styloidectomy among the operated cases of Department of Otolaryngology-Head and Neck Surgery at a tertiary care hospital.

**Methods:**

A descriptive cross-sectional study was conducted among 1,475 who underwent surgery at the Department of Otolaryngology-Head and Neck Surgery in a tertiary care center of Nepal between July 2018 to September 2020 after receiving the ethical clearance from the Institutional Review Committee (Reference number: 0106201802). Convenience sampling was done and data was entered in Statistical Package for the Social Sciences version 20. Point estimate at 95% confidence interval was calculated along with frequency and proportion for binary data.

**Results:**

Among 1,475 patients enrolled in the study, 24 (1.62%) patients (95% Confidence Interval = 0.97-2.26) underwent trans-oral extra tonsillar approach of surgery for Eagle's syndrome during the study duration.

**Conclusions:**

The prevalence of styloidectomy among the operated cases of our study is low in comparison to other studies done in similar settings. Transoral extra tonsillar approach can be considered as a novel approach for surgical removal of the styloid process in Eagle's Syndrome.

## INTRODUCTION

Although there is great variation in the normal length of the styloid process, it is found to be 20-30 mm in the majority of the patients.^1^ When it is longer than 30 mm, it is called elongated styloid process.^1^ An elongated styloid process or calcified stylohyoid ligament causing recurrent throat pain or foreign body sensation, dysphagia, or facial pain is known as Eagle's syndrome.^2-4^

Eagle's syndrome is difficult to identify anatomically with limited clinical understanding.^5^ Elongated styloid process (ESP) has variable incidence i.e., 2-28%- and even lesser (4-10%) are symptomatic.^6^ Surgical removal of the styloid process to its normal limit via extraoral or intraoral technique is regarded as the best option.^7^

This study aims to find out the prevalence of trans-oral extra tonsillar approach of styloidectomy among the operated cases of Department of Otolaryngology-Head and Neck Surgery at a tertiary care hospital.

## METHODS

This is a descriptive cross-sectional study done among 1,475 patients who underwent surgery at the Department of Otolaryngology-Head and Neck Surgery of Kathmandu Medical College and Teaching Hospital, Kathmandu, Nepal between July 2018 and September 2020. Ethical approval was taken from the Institutional Review Committee of Kathmandu Medical College and Teaching Hospital (Reference number: 0106201802). Patients who had undergone surgery in the Department of Otolaryngology-Head and Neck Surgery of Kathmandu Medical College and Teaching Hospital were included and non-operative cases and those who denied surgery were excluded. Informed written consent was taken from the participants. A convenience sampling technique was used. The sample size was calculated by using the formula,

n = Z^2^ × p × q / e^2^

  = (1.96)^2^ × 0.5 × 0.5 / (0.04)^2^

  = 600

where,

n = sample size,
Z = 1.96 at 95% Confidence Interval,
p = prevalence of styloidectomy for maximum sample size, 50% = 0.5
q = 1-p
e = margin of error, 4%

As the sampling technique used was convenient sampling, we have doubled the calculated sample size, which becomes 1200. However, total of 1475 cases were taken in the study.

Detailed history and thorough Ear, Nose, and Throat (ENT) examinations were done including Nasopharyngeal Laryngoscopy (NPL). Pain due to other factors such as temporomandibular, dental, orthopedic, and pharyngoesophageal causes were ruled out. The diagnosis was confirmed with a CT scan. Each side of the neck was taken as a separate entity. Under all aseptic conditions, all operations were performed under general anesthesia.

Under aseptic precautions, nasotracheal intubation was done and a Boyle-Davis mouth gag was applied. Infiltration with 2% xylocaine with adrenaline was given just medial to the palatoglossal fold and anterior tonsillar pillar. A vertical incision ~2 cm long was made at the site of infiltration and the elongated styloid process was felt by palpation. The fibers of superior pharyngeal constrictor muscle were identified and the muscle fibers were split with the help of a blunt dissector. Finger dissection was used to expose the length of the styloid process. The stylohyoid ligament was cut and the styloid tip was engaged in a ring curette and sharp dissection was made superiorly towards the skull base to stripe the periosteum. The styloid process was removed with a bone nibbler and hemostasis was done. The mucosal incision was closed with one or two absorbable sutures. If the elongated, contralateral styloid process was removed similarly in the same setting. The sterile measuring tape was used to measure the length of the styloid process. They were evaluated in terms of duration of surgery, bleeding, post-operative pain, trismus, post operative infection, dysphagia, weakness of any nerve/sensory disturbances, and remission of symptoms. Visual analog score (0-10) was used to assess pain and dysphagia subjectively. The data were entered into Microsoft Excel and analyzed using the Statistical Package for the Social Sciences (SPSS) version 20. Point estimate at 95% confidence interval was calculated along with frequency and proportion for binary data.

## RESULTS

Among the enrolled 1,475 patients, 24 (1.62%) patients (Confidence Interval = 0.97-2.26) underwent trans-oral extra tonsillar approach of styloidectomy among the operated cases at Department of Otolaryngology-Head and Neck Surgery at a tertiary care hospital. Among the 24 patients, 10 (41.7%) were males and 14 (58.3%) were females with the average age being 38 years.

The length of the styloid process was found to be 36 mm (32 - 46 mm). Post-operative scores were consistently lower after styloidectomy across all individual symptoms. Dysphagia showed the most significant improvement from pre- to post-scores. Surgeries were uneventful and follow-up revealed patients to be symptom-free after surgery.

The average operative time was 32 minutes (25-45 minutes). Postoperatively, three patients experienced moderate pain, trismus, and mild dysphagia in the first week. Two patients developed wound dehiscence at the suture site which healed secondarily. No other intra or post-operative surgery-related side effects like bleeding, retropharyngeal infection, or airway edema were observed. Wound healing was on time and there was no postoperative infection. No paresthesia of any nerve or significant fibrosis in the intraoral scar was noted.

## DISCUSSION

Although styloid process elongation is not uncommon, true Eagle's syndrome is a rare disease. The majority of the patients with the elongated and mineralized styloid processes are asymptomatic and require no treatment. When symptoms do exist, the severity of symptoms is unrelated to the length or extent of the mineralization process.^8^ As a result, diagnosing Eagle's syndrome can be challenging and the differential diagnosis should include all the conditions causing cervicofacial pain including trigeminal, sphenopalatine, glossopharyngeal neuralgias, myofascial pain, mastoiditis, dental pain, chronic tonsillitis, pharyngitis, submandibular sialadenitis, pharyngeal foreign body, neoplasia, and migraine.^9^

The primary diagnostic guide for Eagle's syndrome is the patient's medical history. Validating the diagnosis involves palpation of the lateral tonsillar fossa, infiltration of local anesthesia into the tonsillar fossa combined with the radiological examination.^9^

Eagle's syndrome can be treated conservatively or surgically or both. Reassurance, analgesics, and local corticosteroid or anesthetic administration are all options for conservative treatment, but surgical shortening is the most rewarding and effective way to alleviate symptoms.^8,9,10^ Surgical management of ESP has been defined using a variety of transoral and extraoral cervical approaches, each with its own set of benefits and drawbacks.^9^ A noteworthy comparison between intraoral and extraoral surgical approaches was made by Strauss M, et al.^11^ and Chase DC, et al.^12^

Our present study shows that transoral, extra-tonsillar approach for styloidectomy is safe, easy to perform, quick, avoids external scar as well as extensive fascial dissection. Moreover, the recovery time of this procedure was short. Cai Y, et al.^13^ reported that postoperative pain after tonsil sparing styloidectomy was significantly lower after one week postoperatively.

Three of our patients experienced moderate pain and trismus in the first week post-operatively, who underwent bilateral styloidectomy in the same setting. Since intraoral approaches may cause transient edema at the operation site, submandibular, and retromandibular regions, these brief post-operative complications can be considered common.

In our study, there were no major intraoperative or postoperative complications and there was complete remission of symptoms of all the patients in 6 months follow up. Raychowdhary R, et al.^6^ also described an intraoral, extra-tonsillar approach without any complications.

## CONCLUSIONS

The prevalence of trans-oral extra tonsillar approach of styloidectomy among the operated cases at the Department of Otolaryngology-Head and Neck Surgery is low in comparison to other studies done in similar settings. The most effective treatment of Eagle's syndrome is shortening or removal of the styloid process. A transoral, extra-tonsillar approach is a novel approach in terms of safety and adequacy to treat patients with a clinically and radiologically approved Eagle's syndrome without requiring the need for tonsillectomy.
